# A study of the layout planning of plant facility based on the timed Petri net and systematic layout planning

**DOI:** 10.1371/journal.pone.0239685

**Published:** 2020-09-28

**Authors:** Hanwen Liu, Xiaobing Liu, Lin Lin, Sardar M. N. Islam, Yuqing Xu

**Affiliations:** 1 School of Economics and Management, Dalian University of Technology, Dalian, Liaoning, China; 2 CRRC Dalian R&D Co., Ltd., Dalian, Liaoning, China; 3 Institute for Sustainable Industries & Liveable Cities, Victoria University, Melbourne, Australia; University of Defence, SERBIA

## Abstract

The purpose of this research is to solve the problems of unreasonable layout of the production plant, disorder of the logistics process, and unbalanced production line in discrete manufacturing plants. By analyzing the production process and characteristics, the timed Petri net model is constructed according to the function and connection of each production unit, which is then used to generate a FlexSim simulation model of the production plant logistics system with a simulation software. Therewith the FlexSim simulation model is used to simulate the original layout of the plant, and to analyse the simulation data synthetically to put forward an improvement strategy. Combined with the use of the systematic layout planning method to analyze the overall layout of the plant and logistics relations, we infer the relevant drawings between the production units and determine the improved layout of the facilities. Finally, by comparing the before and after improvement simulation results, it is verified that the combination of timed Petri nets and systematic layout planning is effective to ameliorate the layout of the plant facilities and the logistics system. This method makes up for the factors that traditional methods have not considered, achieves the goal of reducing the cross circuitous route of the plant and the idle rate of equipment, and improving the efficiency of production.

## Introduction

With the increasing degree of automation in China’s discrete manufacturing industry, the potential for improving the efficiency of processing procedures to reduce costs has been consequently narrowing. As the result the key to enterprise competition has gradually shifted to new areas, such as logistics technologies that is closely related to production [1]. Facility planning, as an important part of the improvement of the production logistics system, refers to meeting the necessary constraints in a certain production environment, and determining the reasonable layout form and location of the equipment according to the production goals [2]. For some large-scale equipment products, the transfer time between processes is long and the transfer costs are high. Wang and He shows a reasonable layout of the plant facilities can effectively improve the turnover rate of work in progress and parts, shorten the production cycle, and reduce the transfer cost by approximately 30% [3]. It has been widely used in manufacturing, medicine, education, and other fields since being proposed by Richard Muther in 1961. The systematic layout planning (SLP) method calculates the logistics between the production units on the basis of analyzing the five basic elements that affect the effectiveness of the system: P (product), Q (output), R (process flow), S (auxiliary department), and T (time). Naqvi et al. use the improved SLP to develop a new layout which reorganizes the production departments of multinational companies that produce diversified products to improve production efficiency [4]. Kumar et al. propose a Data Envelopment Analysis (DEA) Charnes Cooper & Rhodes (CCR) model with constant returns to scale (CRS) to implement the Lean-Kaizen method. This method is considered an effective method to improve the quality system in the fastener industry and contributes to industrial lean [5]. Liu et al. propose an alarm window layout method suitable for the diagnosis process. By using the SLP method to improve the human-system interface problem, the safety digital alarm window of nuclear power plant is designed [6]. Benitez et al. use cluster analysis combined with SLP to plan the report area of the radiology department, using medical experts as a clustering variable to re-plan department to improve the performance of the radiology department [7]. Tarigan et al. propose a ranked positional weight method combined with SLP to modify the production line of wood production and the handling track of material movement [8].

However, the traditional SLP method may be affected by the subjective experience and knowledge limitation when drawing the correlation diagram of the position of the operating unit. Therefore, the final layout design scheme cannot verify its scientificity and rationality. With the development of computer technology, an increasing number of studies have used SLP and simulation software, such as Flexsim [9–12], Witness [13], and Quest [1], to conduct research objects optimization, improvement and case verification. The use of computer technology to help generate new layouts has been valued by scholars. In order to predict the maximum energy of photovoltaic modules more accurately, Stojčić et al. establish a model based on the principles of the fuzzy logic and artificial neural networks and integrate it into the Adaptive Neuro Fuzzy Inference System (ANFIS). Using PVsyst software for sensitivity analysis, they have obtained better prediction results than mathematical models [14]. Sremac et al. propose that based on the input and output data of the research object, a hybrid method of artificial intelligence ANFIS is used to establish a model for the decision of economic order quantity. Then use simulation to conduct sensitivity analysis to verify the effectiveness of the model for different types of goods in the supply chain management [15]. Korde and Sahu conclude that with the assist of the SLP method, the layout of the manufacturing automotive parts plant is organized and analyzed. The results can be verified by using simulation to derive a new layout [16]. Deshpande et al. use Computerized Relative Allocation of Facilities technology (CRAFT) to improve the layout of manufacturing plants. It is proposed the use of Automated Layout Design Program (ALDEP) to reduce costs [17]. Sharafati et al. propose a hybrid ANFIS as a predictive model. Then the error index is used to run the simulation model to estimate the scouring depth downstream of the sluice gate to manage the irrigation system [18]. Suhardini et al. simulate material handling cost and processing time of Material Handling Evaluation Sheet (MHES) to obtain new planning layout evaluation and program selection, which reduced the material handling cost of building material manufacturers [19]. Simulation technology can simulate the actual operation of a logistics system and test the performance of the system with given. It can generate simulation reports including status characteristics (idle, blockage, etc.), the number of inputs and outputs, and the maximum waiting time to identify bottleneck processes and resources, and facilitate the selection of layout schemes and the verification of effects.

In recent years, there have been some methods describing the modeling of production logistics systems. Among them, the Petri net is proposed by Dr. Petri in 1962 has the characteristics of simple, easy-to-understand graphics, and strict and accurate mathematical functions [20]. It has clear advantages in describing the concurrency, conflict, resource sharing, and other uncertain situations of complex and dynamic discrete production systems [21]. Guo et al. propose an adaptive collaborative timed colored Petri net model of the production logistics system in the internet environment and demonstrated that the method is superior to the events-driving method [22]. Considering the movement path of the robot between base stations, Al-Ahmari et al. propose the use of time-delay petri net (TdPN) to optimize the robot motion controller in the robot scheduling problem, and demonstrated the effectiveness through a mathematical model [23]. Yianni et al. use Petri net to build model to inspect different modules. Based on the model, establish a powerful framework for managing railway bridge asset portfolios [24]. Uzam et al. propose a think-globally-act-locally method with weighted arcs (TGALW) for calculating liveness-enforcing weighted arcs in the general Petri net model, which is used to monitor the activity in the flexible manufacturing system [25]. Mejía et al. establish a combination model of colored and ordinary petri net to solve the integration and collaborative project management in the animation and video game (A&V) industries through the Graph Search algorithm [26].

Many international scholars have studied the setting layout optimization based on the SLP method and the modeling of production logistics system based on the Petri net theory, which have applied the simulation software to specific practical cases to solve the problem of facility planning. However, as the research continues to deepen, the issues involved become more complex. In the literature that has been consulted, it is found that these two parts are relatively isolated. This study uses a large-scale world-class wind power bearing manufacturing plant as the research object. Starts from three research aspects of modeling, improvement, and simulation. Firstly, the bearing production process model and logistics timed delay Petri net (TdPN) model are established through the analyses of the production process and logistics process of the research object. The validity of the dynamic behavior of the logistics system is verified by a mathematical model constructed on Petri net. Secondly, the SLP method is used to analyze the logistics intensity between each operation unit to obtain the improved layout. At last, a FlexSim visual simulation model is constructed. Based on the verification results of the Petri net, and under the premise of ensuring the validity of the simulation model, each processing procedure and equipment usage in the original layout and the new layout obtained by the SLP method are simulated and analyzed to determine if a bottleneck in production is an issue. In addition, improvement measures and opinions are proposed to make the plant logistics smoother and maximize the value of the plant logistics system.

### Situation and problem description

The research subject is Wafangdian Bearing Company Limited which is a large-scale wind power manufacturing company located in Dalian, Liaoning, China. Data is collected without special permit. It encompassed an area of 14,592 square meters, of which the plant area was 76 meters long from north to south and 192 meters wide from east to west. There are nearly 500 managers and workers at all levels of the factory, working in three shifts. The company currently has 30 different types of products in progress for rotating pivot bearing of large-scale wind power turbine, such as spindle bearing, yaw bearing, pitch bearing, speed increaser bearing, drive reducer bearing and generator bearing, etc. With an annual production capacity of about 15,000 sets. The company’s current annual order is about 8,000 sets, with a capacity utilization rate of only 53%. One-third of the equipment has been idle for a long time, but the workshop space has been saturated, and excess equipment has caused detours in the workshop. The investigation find that in the initial stage of the factory establishment, only the bearing inner and outer ring processing production, assembly and anti-corrosion processes are carried out in other plants. After the company’s merger planning, the factory needs to complete all the processes from forging to machining, heat treatment, assembly, and anti-corrosion, among others. The production of parts and components directly affect the later assembly process. It is a typical production workshop with a mix of machining and assembly processes. The merger of the factories makes the layout of the production units very unreasonable, the logistics routes of the workshops are not smooth, and many long-distance handling activities are required. Also, unsatisfactory production rhythms and uneven production lines increase the flow of materials and logistics costs in the plant. The area of each production unit and the number of pieces of equipment are shown in Table 1.

**Table 1:** Workshop/Warehouse area and number of equipment.

No.	Operating name	Purpose	Area / m2	Equipment Quantity
**1**	Forgings workshop	Storage of inner and outer ring blanks forgings	20 *18	
**2**	Lathe machining Workshop	Supporting area for hard turning and grinding (hard turning raceway, grinding raceway)	32*20 + 32*18	18
**3**	Drilling workshop	Drilling	32*28	20
**4**	Hot / gear working workshop	Heat treatment, gear processing	32*34	9
**5**	Measurement workshop	Quality item point size measurement	32*22	2
**6**	Precision lathe processing workshop	Raceway, finishing face, outer diameter, inner diameter, chamfer, etc.	34*28+34*16	40
**7**	Assembly workshop	Assembly of inner and outer rings	16*28	15
**8**	Anti-corrosive workshop	Anti-corrosive treatment	30*64	4
**9**	Work in process workshop	Stored in process, semi-finished product	16*28	
**10**	Auxiliary materials warehouse	Storage of tooling, auxiliary materials, and consumables	34*18	
**11**	Quality inspection workshop	Inspection process and finished product	30*8 + 32*8 + 16*14	
**12**	Packaging workshop	Packaged product	36*14	1
**13**	Finished products warehouse	Storage of finished products	12 *18	
**14**	Office building	Production scheduling, purchasing department, quality department, information department, etc.	152 *8	18

**Fig 1:**
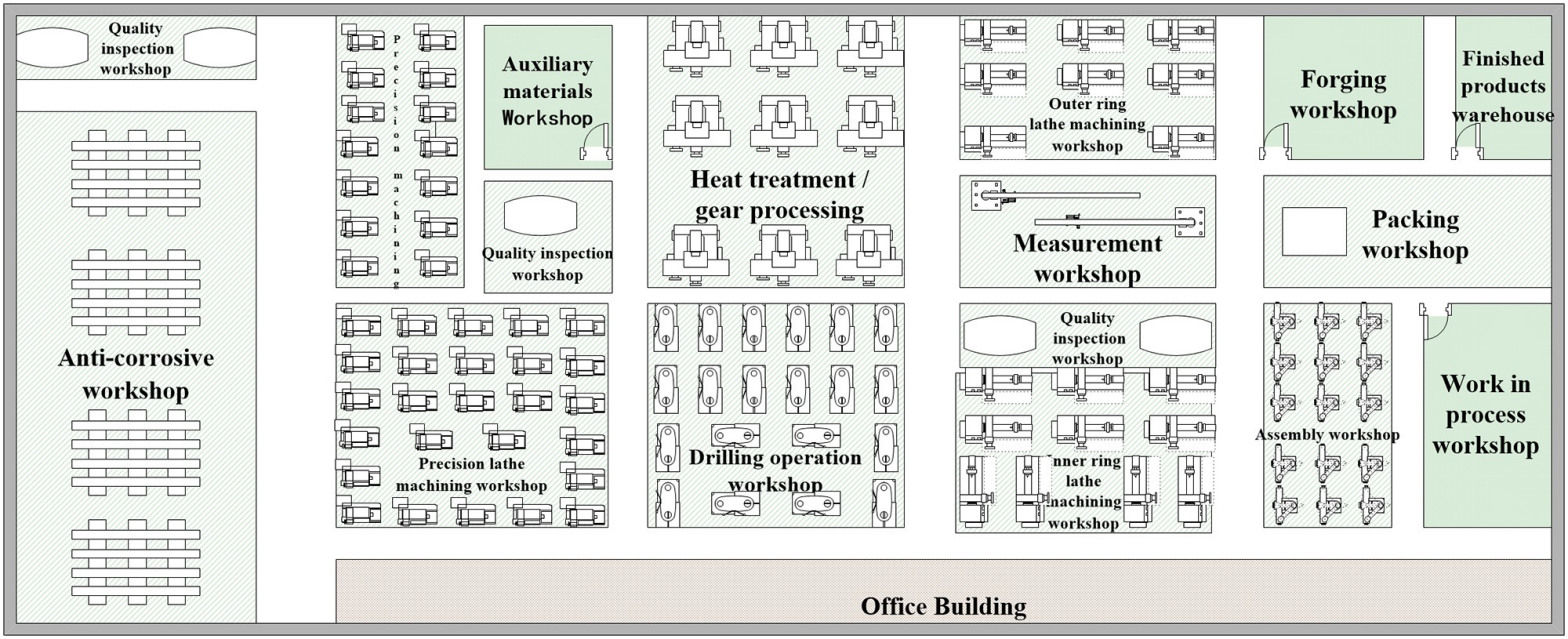
Original layout of the plant.

Based on the data collected from the plant and Table 1, the original layout of the plant is shown in Fig 1.

Facing the fiercely competitive market, the company adopts the one-piece production model, which meets customer needs and strengthen the quality traceability. However, it is not enough to win market orders based on high quality to change the company’s status quo, reasonably arrange plant facilities, and improve equipment utilization rate. Reducing workshop logistics costs and achieving lean production management of enterprises are necessary means as well.

## Methods

**Fig 2:**
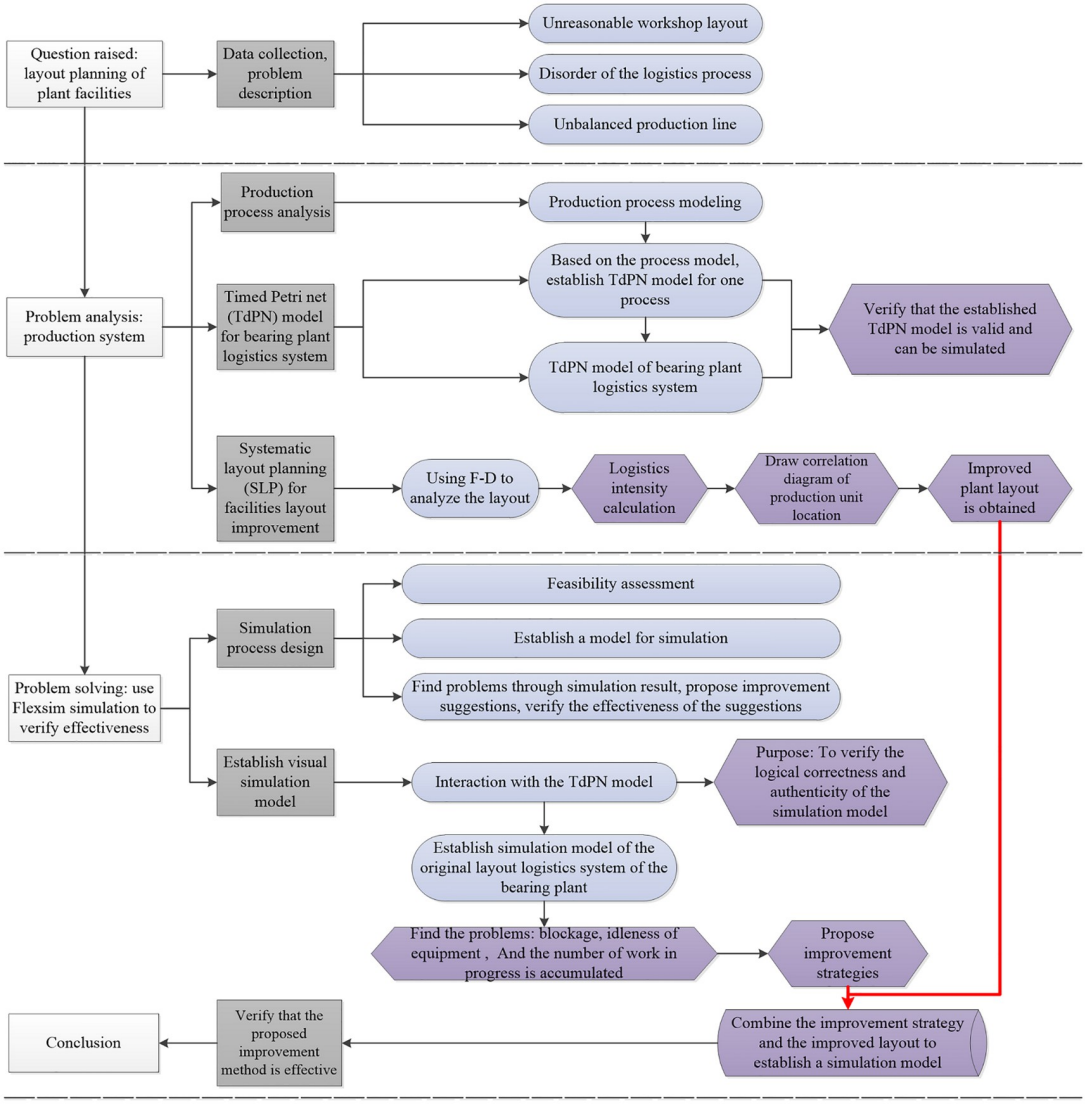
The roadmap of this paper.

For review this paper, the rest structure organization as follows. The bearing production process is analyzed to establish the TdPN model for the plant logistics system. After that, an improved layout of the plant is proposed based on SLP. Afterwards, a simulation model of the original layout is established by the TdPN model and FlexSim software interaction, then the improvement strategies are proposed. Lastly, combined the improved layout with proposed strategies, the improvement simulation model is established to verify the effective. A roadmap of the overall framework, analysis methods and models gives in Fig 2.

### Modeling of plant production process and logistics TdPN model

The plant production system is a typical discrete event system that triggers the occurrence of events with events. Petri net is a network system model used to describe the combination of physical processes and systems which is an effective tool for discrete time modeling. It describes not only the structure, but also the dynamic behavior of the system. Methods hierarchical description and analysis of the properties of complex discrete systems are performed, and refinement is gradually achieved [27]. The basis of the bearing production TdPN model is the production process model. Taking spindle bearing as an example, the production process is analyzed and improved to lay the foundation for the next modeling and simulation.

#### Analysis of bearing production process

**Fig 3:**
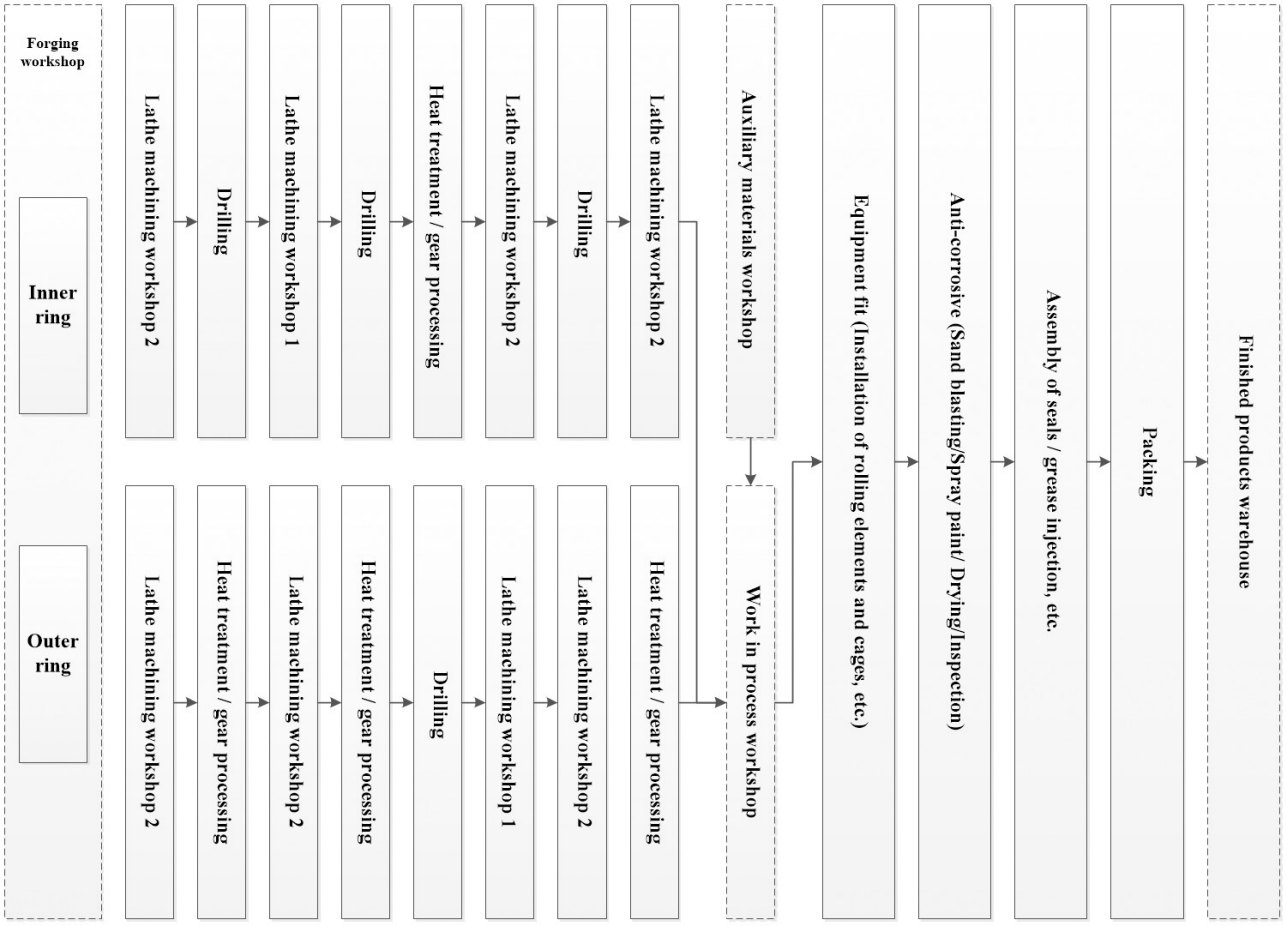
Model of bearing manufacturing process.

Large-scale wind power bearings are mainly composed of four main parts which are inner ring, outer ring, rolling body and cage, and other auxiliary parts, such as seals, dust covers, and rivets. Bearing production mainly includes the two processes of inner and outer ring processing and fitting assembly. The inner and outer ring forgings are respectively processed through lathe machining, heat treatment/gear processing, drilling, and other testing auxiliary processing to form semi-finished products of the inner and outer rings. The semi-finished products of the inner and outer rings that have undergone grinding and other processing techniques must be cleaned and dried before final assembly. The following steps are sorting into sets, installing rolling elements, protective rubber ropes, seals, screws, and so on. After that, sandblast and spray-paint for corrosion protection and drying processes are followed. Finally complete removal the semi-finished seals, installation the seals, mounting screws, grease and other assembly and packaging processes to form the finished bearing products, which stores in the finished product warehouse. In actual production, the process as the basis for the standard process of production and plan scheduling is very detailed. For example, lathe machining includes ordinary lathe machining (lathe machining 1) and precision lathe machining (lathe machining 2). The main processes are shown in Table 1. To facilitate the study, the modular modeling of its process according to the processing flow is shown in Fig 3.

#### Timed Petri net (TdPN) model for bearing plant logistics system

**Fig 4:**
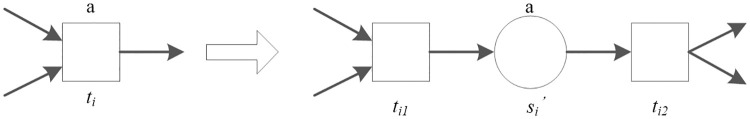
Non-transient transitions replaced by time value bases.

**Fig 5:**
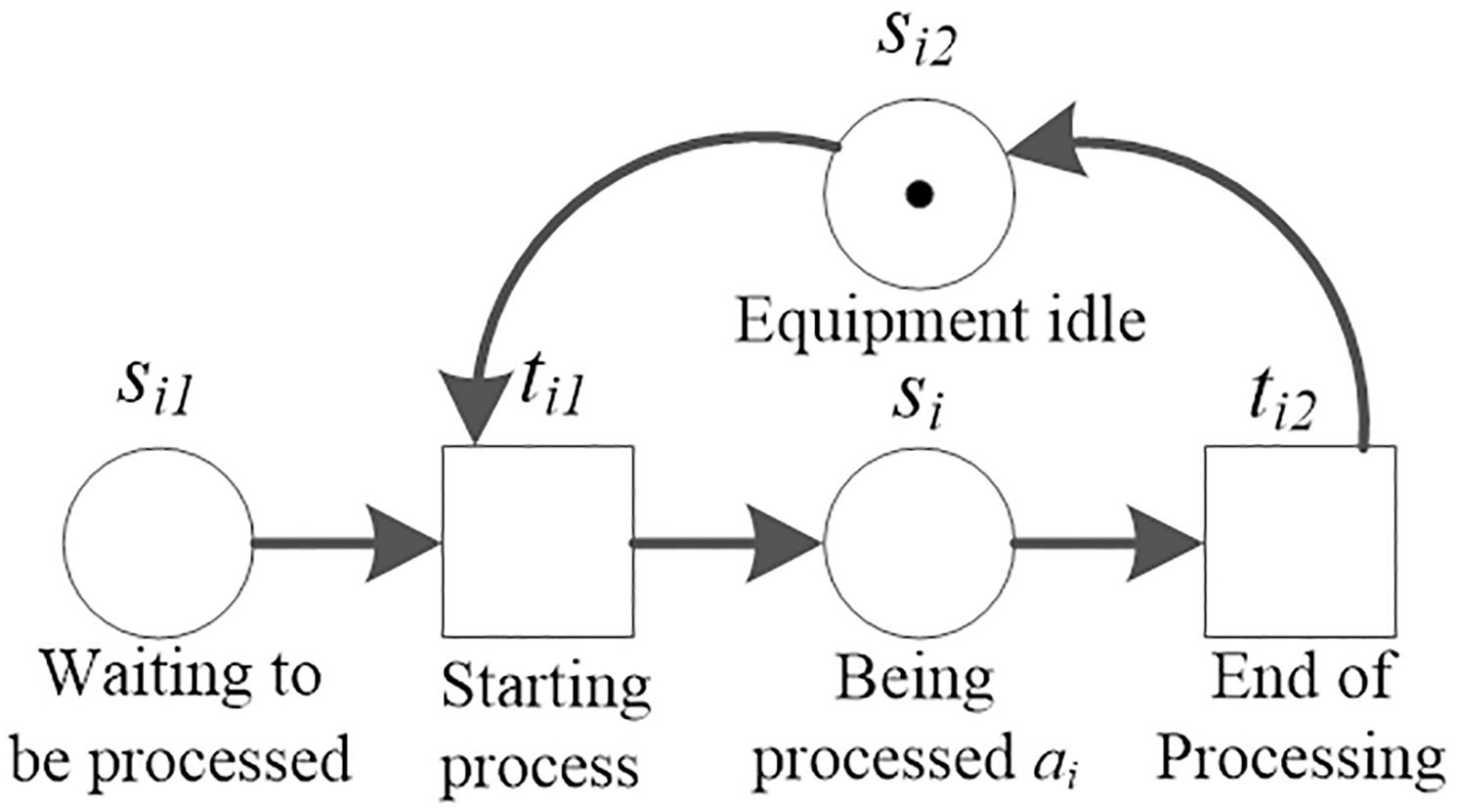
Petri net model of one process.

*TdPN definition*. TdPN is first proposed by Ramchandani to analyze the performance of asynchronous concurrent systems [28]. TdPN is a 5-tuple ∑ = (*S*, *T*; *F*, *M*, *DI*), where (*S*, *T*; *F*, *M*) is a prototype Petri net. *S* = {*S*_1_, *S*_2_, ⋯, *S*_*n*_} is a set of finite place of ∑, *T* = {*T*_1_, *T*_2_, ⋯, *T*_n_} is a set of finite transitions of ∑; *F* is an ordered pair set consisting of an *S* element and a *T* element in ∑, which is called the flow Relation of ∑ Relation; *M*: *S* → {0, 1, 2, ⋯} is called a ∑ mark, the mark *M*_0_ (*s*) represents the distribution of tokens in the warehouse in the initial state. *R*(*M*_0_) represents the set of all of the states that may occur during the system operation, and DI is the definition time function on transition set *T*, that is, DI: *T* → *R*_0_. When a TdPN is represented graphically, *S*_*n*_ is drawn as a small circle, *T*_*n*_ is drawn as a small rectangle, *F* is drawn as a directed edge and exists only between the small circle and the small rectangle, and *M*(*s*) is represented by a black dot in *S*_*n*_. The drawing of this description can refer to Fig 5. For *t* ∈ *T*, DI(*t*) = *a* means that the occurrence of transition *t* requires *a* unit time to complete. That is, when a mark *M* satisfies *M*[*t* >, the transition *t* can occur immediately, but the occurrence of *t* ends only after *a* time units has elapsed. The above definition of TdPN violates the transient principle of transition. Therefore, in TdPN, for each non-transient transition can be replaced by a sub-net composed of two transient transitions and a time-valued place (Fig 4).

*TdPN model*. The production, processing, and assembly of bearings can be divided into several processes. According to the connection relationship between the processes and the duration of each process, combined with the basic principles of Petri net, a TdPN model of the bearing production process is constructed. The connection of three plants and two transitions represent that the work-piece completes one process and is ready to enter the next process (Fig 5), where *t*_*i*1_ represents the start of process *i*, *t*_*i*2_ represents the completion of process *i*, and place *s*_*i*_ means that process *i* is processing, and assigns a time value *a*_*i*_ to storehouse *s*_*i*_, which means that from *t*_*i*1_, at least *a*_*i*_ time unit must elapse before *t*_*i*2_ can occur. *s*_*i*1_ means that the workpiece is ready for processing, and *s*_*i*2_ means equipment for processing *s*_*i*_ it is usable or not.

**Fig 6:**
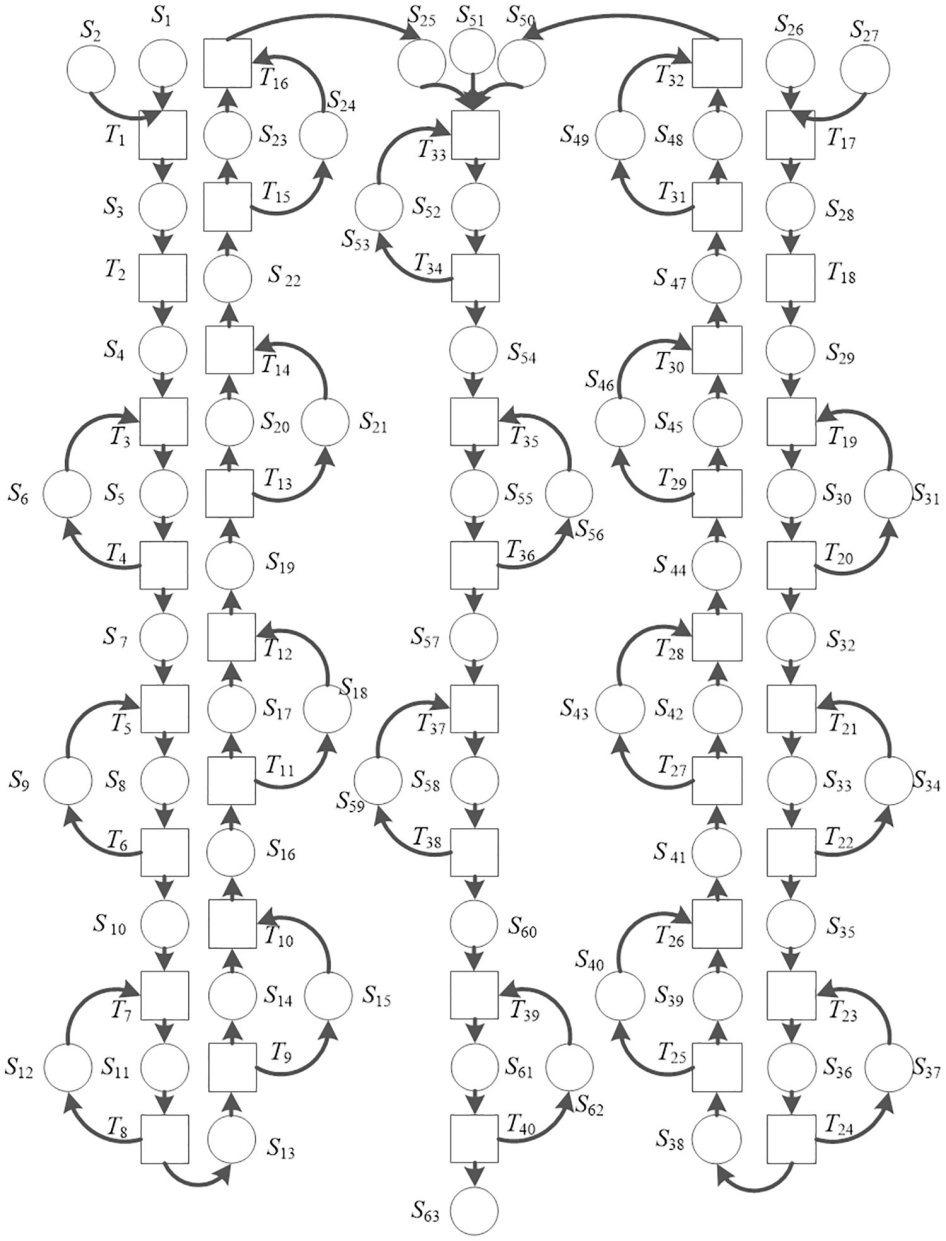
TdPN model of bearing plant logistics system.

According to the analysis of the bearing production process outlined above, a TdPN model is established for the production logistics system of the bearing plant. The establishment of the TdPN has been a guiding role for logistics analysis, plant construction, facility layout, and actual production. Fig 6 is a TdPN model of the logistics system for the processing and assembly of the inner and outer rings of a bearing plant. This logistics system model is composed of an inner circle production line, an outer circle production line, and packaged wiring.

The meanings of the symbols (*S*, *T*) in Fig 6 explain in Tables 2 and 3 which are the product processing time, waiting for status and equipment status (Table 2), and the product processing status (Table 3).

**Table 2:** Implication of the workshop.

Workshop	Implication	Time	Plant	Implication	Time
*S*_1_, *S*_26_	Inner and outer ring forgings waiting to be processed		*S*_25_, *S*_51_	Inner and outer ring waiting for fitting assembly	
*S*_2_, *S*_9_, *S*_21_, *S*_27_, *S*_34_, *S*_43_	Precision lathe processing equipment is idle		*S*_53_	Bearing assembly equipment idle	
*S*_3_, *S*_28_	Precision lathe machining of inner and outer ring forgings	280,120	*S*_50_	Bearing assembly accessories ready	
*S*_4_, *S*_10_, *S*_22_, *S*_38_	Inner and outer rings waiting for hot / tooth processing		*S*_52_	Bearing assembly	70
*S*_6_, *S*_12_, *S*_24_, *S*_40_	Hot / tooth processing equipment is idle		*S*_54_	Bearing waiting for corrosion protection	
*S*_5_, *S*_11_, *S*_23_, *S*_39_	Inner and outer ring heat / tooth processing	140,780,1945,140	*S*_56_	Bearing anti-corrosion equipment is idle	
*S*_7_, *S*_19_, *S*_32_, *S*_41_	Inner and outer rings waiting for precision lathe processing		*S*_55_	Bearing anti-corrosion processing	2215
*S*_8_, *S*_20_, *S*_33_, *S*_42_	Inner and outer ring precision lathe machining	84,75,140,60	*S*_57_	Bearings waiting to be fitted with seals, etc.	
*S*_13_, *S*_29_, *S*_35_, *S*_44_	Inner and outer ring waiting for drilling		*S*_59_	Bearing assembly seals / grease injection equipment are idle	
*S*_15_, *S*_31_, *S*_37_, *S*_46_	Inner and outer ring drilling equipment is idle		*S*_58_	Processing of bearing assembly seals / grease injection, etc.	225
*S*_14_, *S*_30_, *S*_36_, *S*_45_	Machining of inner and outer rings	635,110,70,198	*S*_60_	Bearings waiting to be packed	
*S*_16_, *S*_47_	Inner and outer rings waiting for general machining		*S*_62_	Bearing packing equipment idle	
*S*_18_, *S*_49_	Inner and outer circle ordinary lathe equipment is idle		*S*_61_	Bearing packaging processing	45
*S*_17_, *S*_48_	Inner and outer ring ordinary lathe machining	270,280	*S*_63_	Bearing finished product storage	

**Table 3:** Implication of shift.

Shift	Implication	Shift	Implication	Shift	Implication
*T*_1_, *T*_5_, *T*_13_, *T*_17_, *T*_21_, *T*_27_	Start of precision lathe machining of inner and outer rings	*T*_11_, *T*_31_	Start of ordinary lathe machining of inner and outer rings	*T*_37_	Start of assembly of bearing accessories
*T*_2_, *T*_6_, *T*_14_, *T*_18_, *T*_22_, *T*_28_	End of precision lathe machining of inner and outer rings	*T*_12_, *T*_32_	End of ordinary lathe machining of inner and outer rings	*T*_38_	End of assembly of bearing accessories
*T*_3_, *T*_7_, *T*_15_, *T*_25_	Start of Heat / tooth machining of inner and outer rings	*T*_33_	Start of bearing sleeve assembly	*T*_39_	Start of bearing packaging
*T*_4_, *T*_8_, *T*_16_, *T*_26_	End of Heat / tooth machining of inner and outer rings	*T*_34_	End of bearing sleeve assembly	*T*_40_	End of bearing packaging
*T*_9_, *T*_19_, *T*_23_, *T*_29_	Start of drilling of inner and outer rings	*T*_35_	Start of bearing corrosion protection		
*T*_10_, *T*_20_, *T*_24_, *T*_30_	End of drilling of inner and outer rings	*T*_36_	End of bearing corrosion protection		

Due to resource shortage or congestion, system operation may be stopped, that is, no changes can be started; such a net is not liveness. Let ∑ = (*S*, *T*; *F*, *M*, *DI*) be a delayed Petri net. If *M* ∈ *R*(*M*_0_) makes ∀t ∈ *T*: ¬*M*[*t* >, then *M* is called a dead marking of ∑. If there is no dead mark in ∑, then ∑ is called non-dead. In Fig 6, the *M* = {*S*_1_, *S*_2_, ⋯, *S*_63_}^*T*^ is identified, where *S*_1_, *S*_26_, *S*_51_ is the input places and *S*_63_ is the output place. The specific situation of the bearing plant is analyzed to obtain the initial mark *M*_0_ = {1,1,0,0,0,1,⋯,1,1,0,0,0,1,⋯,1,0,0,1,0}^*T*^ of the Petri net. According to the Petri net change rules, *T*_1_ is changed under the initial mark, *T*_17_ has the right to occur. If *T*_17_ occurs, the status of the new system can be determined, that is, the new logo *M*_1_ = {0,0,1,0,0,1,⋯,0,0,1,0,0,1,⋯,1,0,0,1,0}^*T*^. After 120 minutes, the inner ring forging precision lathe machining is completed, *T*_18_ has the right to generate, *M*_2_ = {0,0,1,0,0,1,⋯,0,0,0,1,0,1,⋯,1,0,0,1,0}^*T*^, at this time *T*_19_ has the right to occur. The inner ring drilling is finished after 110 minutes, at this time the outer ring is still undergoing precision lathe machining of forgings. By analogy, the transition set is excited in turn, and *M*_40_ = {0,1,0,0,0,1,⋯,0,1,0,0,0,1,⋯,1,0,0,1,1}^*T*^ is obtained. According to the above analysis, both ∀*M* ∈ *R*(*M*_0_) and ∃*M*’ ∈ *R*(*M*) in the model make *M*‘[*t* >, so the model is reachable, active, and bounded, which is convenient for simulation.

### Bearing plant facility layout and simulation

#### Facilities layout optimization of bearing plant based on systematic layout planning (SLP) method

The SLP method obtains the logistics flow chart of the plant production system by analyzing the logistics process of the manufacturing process. Analyzing the problems in the layout of the plant facilities by using F-D (flow-distance) afterwards, and drawing a correlation diagram of the location of the production unit according to the logistics relationship to improve the plant logistics system. The closeness of logistics intensity between production units is calculated and the degree of precision is analyzed based on the floor plan of a large-scale wind power bearing manufacturer in Fig 1, the distance between production units (approximately the location of each area at the center point), the scale of production (600 monthly production), and the production process (Fig 3).

Assuming that the bearing production system has *n* production units, and the material flow matrix from point *i* to point *j* of every two production units is *Q* = [*q*_*ij*_]_*n*×*n*_. When *i* to *j* has no material flow relationship, that is, *q*_*ij*_ = 0, calculate the distance matrix *D* = [*d*_*ij*_]_*n*×*n*_ according to Fig 1, then the minimum logistics intensity of the entire system is $F=min\sum_{i=1}^{n}\sum_{j=1}^{n}q_{ij}d_{ij}$. According to the collected data and the above formula, a summary table of the bearing plant logistics intensity is calculated (Table 4).

**Table 4:** Summary of the logistics intensity of the production unit of the bearing plan.

	**To**	**1**	**2**	**3**	**4**	**5**	**6**	**7**	**8**	**9**	**Total**
**From**		Forging	Lathe machining workshop 1	Lathe machining workshop 2	Drilling	Heat/Gear	Work in progress	Auxiliary material warehouse	Assembly workshop	Anti-corrosive workshop	
**1**	Forging workshop			1423890							1423890
**2**	Lathe machining workshop 1			1230533	1277542						2508075
**3**	Lathe machining workshop 2				1697515	745020	2089768				4532303
**4**	Drilling		1552618	848757		339503					2740878
**5**	Heat/Gear			1490041	1433185		836025				3759252
**6**	Work in progress								452424		452424
**7**	Auxiliary material warehouse								55567393		55567393
**8**	Assembly workshop									66319272	66319272
**9**	Anti-corrosive workshop								66319272		66319272
**Total**		0	1552618	4993222	4408243	1920548	2089768	0	122339089	66319272	203622762

Because there are a lot of logistics data in large bearing plant, five grades are introduced in the SLP method to describe the logistics intensity (Table 5). When analyzing the logistics intensity, the movement sequence and movement volume of the logistics objects are determined, followed by taking the movement volume of the logistics objects within a certain period as the logistics intensity.

**Table 5:** Classification of logistics intensity level.

Logistics intensity level	Symbol	Percentage of workload	The percentage of logistics of total amount	Score	Line	Logistics intensity level	Symbol	Percentage of workload	The percentage of logistics of total amount	Score	Line
Ultra-high logistics level	A	10%	40%	4	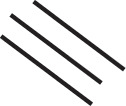	General logistics intensity	O	40%	10%	1	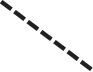
Extra high logistics intensity	E	20%	30%	3	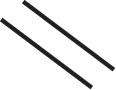	Negligible intensity	U			0	
Extraordinary logistics intensity	I	30%	20%	2	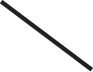						

The five levels of intensity are A (absolutely important), E (extremely important), I (Important), O (ordinary important), U (unimportant). Table 5 indicates that the more lines, the stronger the logistics intensity.

According to the classification criteria and the logistics intensity in Table 4, the logistics intensity levels of each production unit can be analyzed which is shown in Table 6. Each pair of units in Table 6 is calculated based on the data in Table 4.

**Table 6:** Logistics intensity from production unit to production unit.

No.	Units	Logistics intensity level	Grade	No.	Units	Logistics intensity level	Grade	No.	Units	Logistics intensity level	Grade
**1**	1–3	1423890	**O**	**5**	3–5	2235061	**I**	**9**	6–8	452424	**O**
**2**	2–3	1230533	**O**	**6**	3–6	2089768	**I**	**10**	7–8	55567393	**E**
**3**	2–4	2830160	**I**	**7**	4–5	1772688	**O**	**11**	8–9	132638544	**A**
**4**	3–4	2546272	**I**	**8**	5–6	836025	**O**				

**Fig 7:**
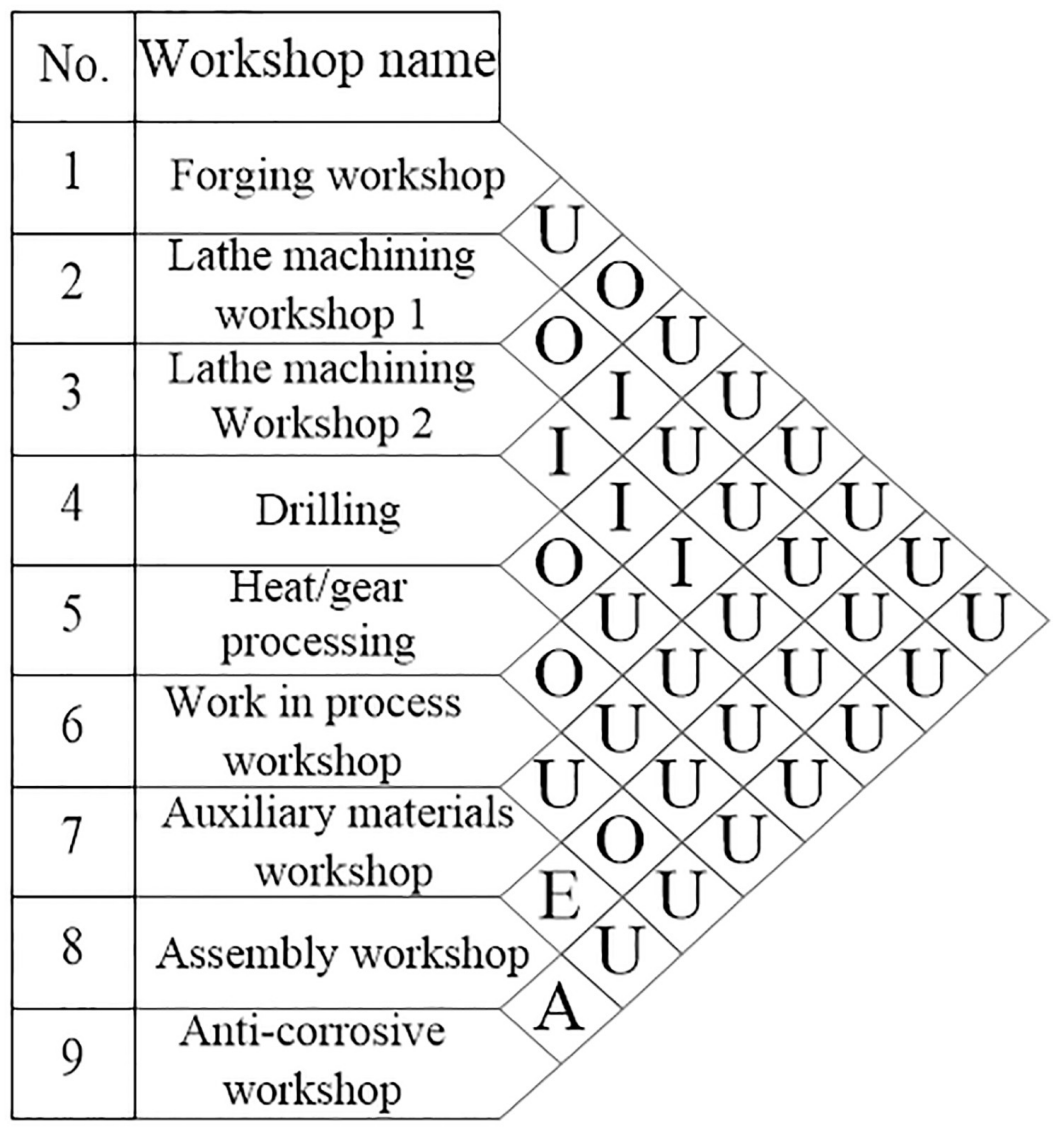
Production unit correlation diagram.

Based on the vertical order of the workshops listed in Table 4, the logistics intensity grade for each production unit is determined and presented in Table 6. Hence, the correlations of the production units are classified in Fig 7.

Due to the large number of interrelations between production units in the bearing plant, the closeness score of each production unit needs to be calculated according to Table 5 and Fig 7, in order to draw a plant layout map. A higher score indicates that the production unit is closer to the center of the layout map. In comparison the production unit with a lower score should be closer to the edge of the layout. Therefore, the plant is re-arranged according to the order of closeness scores which is lathe machining workshop 2 → assembly workshop → drilling operation workshop → heat treatment / gear processing operation workshop → work in process workshop → anti-corrosive workshop → lathe machining workshop 1 → Auxiliary materials workshop →Forging workshop.

In the location relationship diagram of production units, the production units are represented by the numbers marked in ‘○’, and storage units are represented with ‘△’. The interrelationship between production units uses different types of line connections. Nevertheless, the nature of production units is not necessarily strictly distinguished with this method.

**Fig 8:**
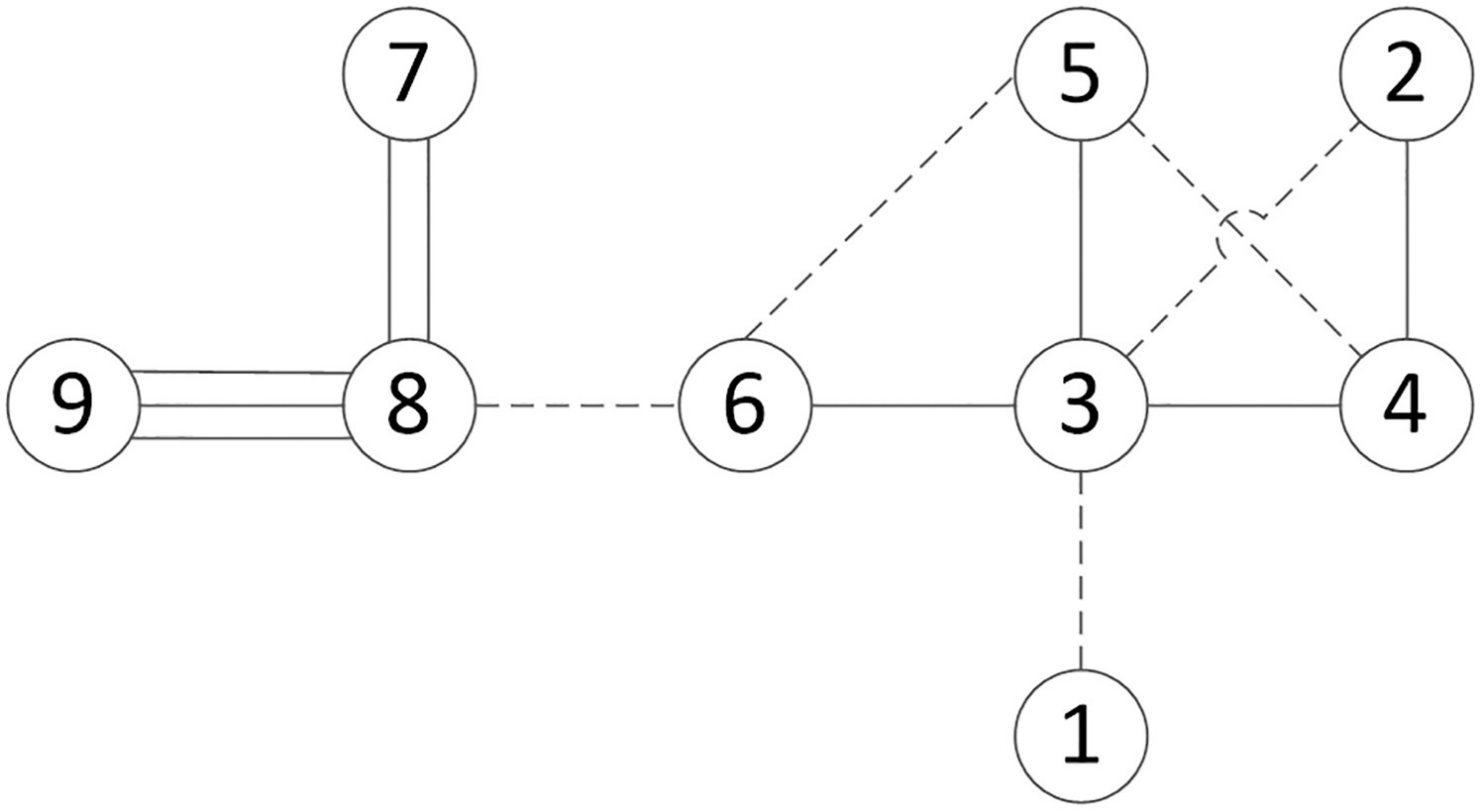
Correlation diagram of production unit location.

For example, the logistics intensity between anti-corrosive workshop (9) and assembly workshop (8) is found to be the strongest (grade A) from Fig 7, which is represented with three lines connecting the two numbers. The numbers 1–9 are represented the workshops name shows in Fig 7. By applying this method to all the workshops, Fig 8 is drawn to show the correlations of the production unit locations.

**Fig 9:**
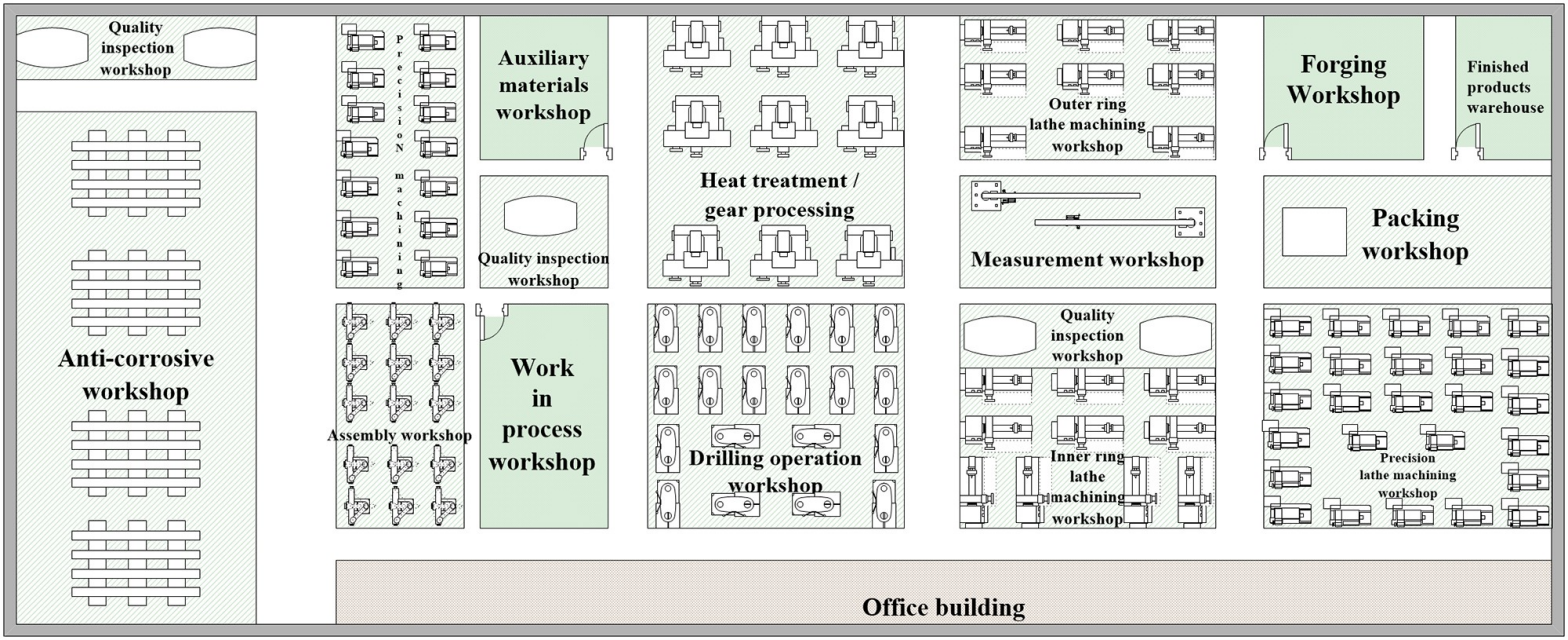
Plant layout after improvement.

By combing with the initial layout of the plant, the location correlation diagram (Fig 8) and the actual situation of the plant, the new plant layout (Fig 9) is drawn after the interchanging of assembly workshop, the work in process workshop and the precision lathe processing operation workshop.

#### Simulation of bearing plant logistics system based on TdPN

The above-mentioned TdPN model for the logistics delay of the bearing plant clearly and intuitively described the bearing production process, real-time status, the transition between states, and the logical relationship between processing procedures and resources. However the Petri net could not affect the production system’s efficiency, and resource utilization quantitative evaluation and amelioration. Simultaneously, the SLP method is used to obtain the improvement layout map between the production units in the plant, but whether it has more advantages than the original layout plan needs further verification and analysis. Therefore, the entire plant logistics system is simulated with the simulation software which could solve this problem effectively. Simulation data that are closest to the actual state is analyzed to predict the problems that may exist in the production, propose improvement solutions, and verify the effectiveness of the scheme.

#### Simulation process design

**Fig 10:**
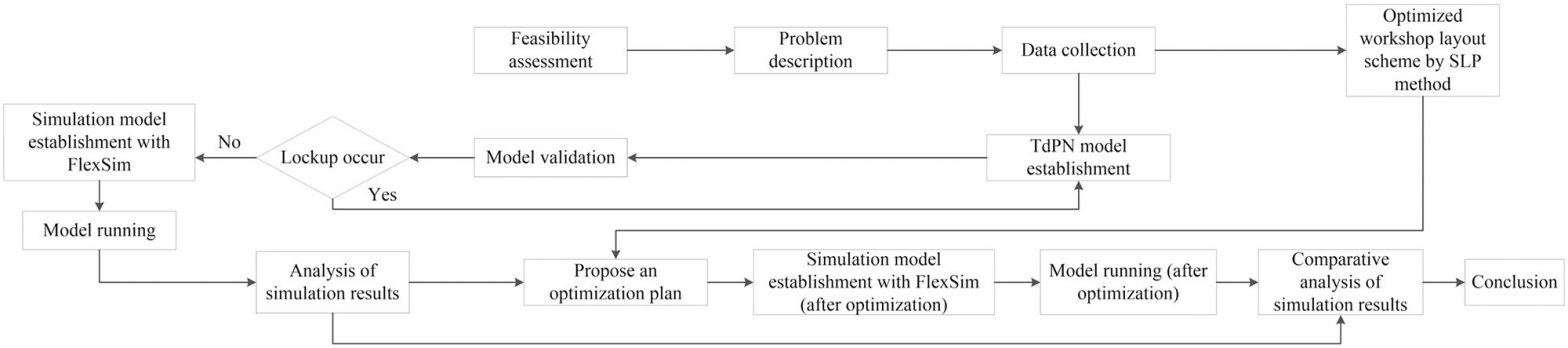
Design diagram of simulation process.

A reasonable production logistics modeling and simulation process is designed for bearing plant production logistics system, which includes three important research stages, the preliminary preparation stage, the modeling and simulation stage, and the analysis and optimization stage (Fig 10).

1. The preliminary preparation stage.
  The work at this stage can be divided into feasibility assessment, problem description and data collection. The feasibility assessment is to evaluate the SLP method of bearing plant layout improvement, Petri net modeling of production logistics system, the simulated problem, and the feasibility of the method. The problem description refers to accurately and comprehensively collecting information on the process of bearing production and the distribution of plant equipment, and analyzing and discovering problems to clarify the tasks of modeling and simulation. Data collection is to collect data on the shift schedule, equipment processing time, production capacity, and area of each workstation in the actual production process.
2. The modeling and simulation stage
  The main work in this stage includes two steps. The first step is establishment and mathematical analysis of TdPN model which precisely describes the nature of the actual production system based on the bearing production process in the preliminary preparation stage. The second step is establishing and running the FlexSim simulation model which is to map the Petri net model into FlexSim software according to certain rules. By setting parameters and adjusting resources, the FlexSim simulation model is generated. Processes can be found in chapters of ‘Timed Petri net (TdPN) model for bearing workshop logistics system’ and ‘establishment of simulation model’.
3. The analysis and improvement stage.
  This stage is to analyze the results of the simulation operation of the bearing production system to find the reason of the entire system problem and production imbalance. Based on the problems found, with the integration of the improved plant layout obtained by SLP method, a specific improvement strategy is proposed. Finally, the proposed method and improvement strategy is verified by running the improvement model and analyzing the simulation results (refers to chapters ‘Facilities layout improvement of bearing plant based on systematic layout planning (SLP) method’ and discussion).

#### Establishment and running of simulation model

The FlexSim software can establish an object-oriented visual simulation model which has a interaction and mapping relationship with the Petri net model. In the Petri net model, the plants represent different types of resources corresponding to the entities in FlexSim. When analyzing process logistics, each step is regarded as a processing unit with only inputs and outputs. The changes in the Petri net correspond to the port of the entity in FlexSim. The activity of the Petri net can be used to verify the correctness of the logic of the FlexSim simulation model, to fully restore the actual production system, and to ensure the authenticity of the simulation model. According to the number of plant equipment, the floor plan of the plant layout and the Petri net model combined with the simulation entity type and meaning, a physical simulation model corresponding to the plant production unit is established, and the parameters of each entity are set. The entity type, entity name, and meaning of each production unit are shown in Table 7. Among them, in order to visually show the physical flow and the unbalance production situation in the bearing plant, temporary storage areas have been added before and after the processing production unit for storing work in progress. The maximum capacity is set to 1000 to avoid the phenomenon that the before and after processes are not connected and unable to work. Finally, in the bearing production process, it is necessary to take the material (inner ring, outer ring, and auxiliary materials) at regular intervals. The raw materials generated by the generator and the time to reach the warehouse to take material are subject to a normal distribution [29]. The processor provides time setting and flow direction setting functions, which indicate the processing time of different processes in production and assembly. Large-scale wind power bearings are large in volume and can only be carried by forklifts one at a time.

**Table 7:** Entity name, meaning and corresponding production unit.

Production unit	Entity type	Meaning	Production unit	Entity type	Meaning
Forgings workshop	Generator 192, 280.	Generate inner and outer ring forgings.	Auxiliary materials warehouse	Generator 1.	Generate rolling bodies, etc.
	Temporary storage area 193, 281	Storage of inner and outer ring forgings		Temporary storage area 2	Storage cage, etc.
Precision lathe workshop processing workshop	Processor 294–298, 215–218, 235–237, 331 etc.	Precision process machining (grinding).	Lathe working area	Processor 194–197, 282–287, 322, 333, 243 etc.	Ordinary process machining (grinding). Temporary storage of work-in-progress before and after ordinary processing of inner and outer rings
	Temporary storage area 241, 232, 217etc.	Temporary storage of work in progress before and after precision machining of inner and outer rings		Temporary storage area 206, 247, 323 etc.	
Hot / gear workshop	Processor 306–308, 345–348, 212, 290 etc.	Heat treatment / tooth processing;	Drilling area	Processor 310–318, 221–223, 207, 208, 203, 199 etc.	Drilling process; Temporary storage of work-in-progress before and after drilling of the inner and outer rings
	Temporary storage area 309, 291, 213 etc.	Temporary storage of work-in-progress before and after heat treatment / tooth processing of inner and outer rings		Temporary storage area 319, 211, 204 etc.	
Anti-corrosive workshop	Processor 9–12.	Anti-corrosive process.	Assembly workshop	Synthesizer 23–34, 5–7.	Equipment process machining. Temporary storage of bearing in process
	Temporary storage area 13	Temporary storage bearing in process		Temporary storage area 35, 8	
Work in process workshop	Absorber 253, 419.	Absorb the work-piece after all the inner and outer ring processing procedures are completed. Storage of finished inner and outer rings	Finished products warehouse	Absorber 246.	Absorb the work-piece after all bearing processing procedures are completed. Storage of finished bearing
	Temporary storage area 252, 352			Temporary storage area 36	

**Fig 11:**
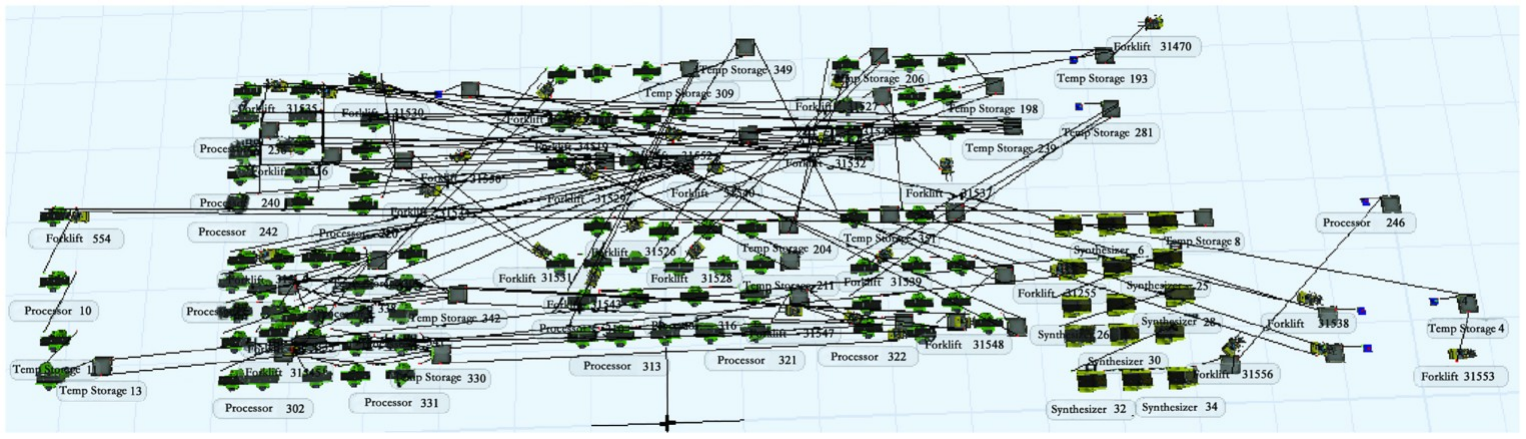
Simulation model of the original layout logistics system of the bearing plant.

According to the status of each entity in Table 7, the production process entities are connected by using the "A" key, while the "S" key is used for forklifts as handling tools. Those two keys indicate the different state transitions and the mutual relationships of the resources. To ensure product quality and reduce the rate of rejects, in actual production, quality inspection is required for each process. A special work area for quality inspectors is set up in the workshop. However, due to the large weight and volume of the product, the quality inspection process is not reflected in the simulation model as it is carried out by the quality inspector to the corresponding station, which does not generate product material flow. The simulation model is established in FlexSim for the initial layout (Fig 11).

After setting up the FlexSim simulation model of the bearing plant production system, the parameters of the simulation processor are set according to the bearing production process flow, equipment processing time, shift calendar etc. Then, the simulation model is run to achieve visualize production dynamics. To better express the performance of the bearing plant production logistics system, the end state simulation method is used in this paper. With the simulation stop time set to 28800, the simulation report data are retrieved after running the simulation model (Table 8). The simulation results in Table 8 can intuitively express information such as the number of items entering and leaving in different devices, the average time of items staying in the device, the device idle time, the device working time, and the device blocking time.

**Table 8:** Initial simulation result.

Class	stats input	stats output	stats stay time	state since	idle	processing	blocked
Processor l94	268	267	60	28766	12746	16020	0
Processor l95	19	19	60	27399	26259	1140	0
Processor l96	267	267	60	28793	12773	16020	0
Processor l97	19	19	60	27459	26319	1140	0
Processor l99	285	285	10	28743	25893	2850	0
Processor 200	285	285	50	28793	14543	14250	0
Processor 201	285	285	5	28798	27373	1425	0
Processor 203	285	284	45	28798	1601S	12780	0
Processor 205	175	174	140	28734	4374	24360	0
Processor 207	174	174	10	28761	27021	1740	0
Processor 208	174	173	40	28761	21S41	6920	0
Processor 210	173	173	20	28659	25199	3460	0
Processor 212	165	164	140	28799	5839	22960	0
Processor 214	163	163	60	28691	18911	9780	0
Processor 221	157	156	153	28683	4815	23868	0
Processor 222	156	156	15	28674	26334	2340	0
Processor 223	156	156	30	28704	24024	4680	0
Processor 224	156	156	20	28735	25615	3120	0
Processor 227	155	155	30	28665	24015	4650	0
Processor 234	87	86	230	28609	8829	19780	0
Processor 235	86	86	80	28707	21827	6880	0
Processor 243	86	85	15	28786	27511	1275	0
Processor 282	156	156	70	28778	17852	10920	6
Processor 283	131	131	70	28438	19268	9170	0
Processor 285	156	155	75	28778	17107	10S50	821
Processor 286	155	154	140	28765	7205	21560	0
Processor 287	131	131	70	28508	19338	9170	0
Processor 288	131	131	140	28648	10308	18340	0
Processor 290	150	149	140	28790	7930	20860	0
Processor 292	149	148	64	28799	19327	9472	0
Processor 297	148	148	20	28693	25733	2960	0
Processor 306	72	71	360	28710	3150	25560	0
Processor 307	71	70	210	28677	13977	14700	0
Processor 308	70	70	210	2S704	14004	14700	0
Processor 310	70	69	260	28738	10798	17940	0
Processor 312	69	69	15	28620	27585	1035	0
Processor 320	69	6S	360	28620	4140	244S0	0
Processor 322	6S	6S	10	28605	27925	6S0	0
Processor 324	6S	6S	10	28699	28019	6S0	0
Processor 333	6S	6S	20	28780	27420	1360	0
Processor 335	67	67	60	28538	24518	4020	0
Processor 343	67	66	240	28607	12767	15840	0
Processor 345	37	36	647	28685	5384	17280	6021
Processor 346	36	35	720	28617	3417	25200	0
Processor 347	35	34	720	28617	4137	24480	0
Processor 348	34	33	210	28617	21687	6930	0
Processor 9	22	21	11S3	28726	3591	6380	18754
Processor 10	21	20	1376	28581	766	6300	21515
Processor 11	20	19	1440	28281	921	27360	0
Processor 12	19	19	150	28431	25581	2850	0

## Discussion

### Analysis and improvement of simulation results

#### Result analysis

This paper mainly studies the bottleneck of the production logistics system of the bearing plant, improving of the equipment layout of the plant. The equipment idle rate and the number of work in process are analyzed according to the characteristics of bearing production.

**Fig 12:**
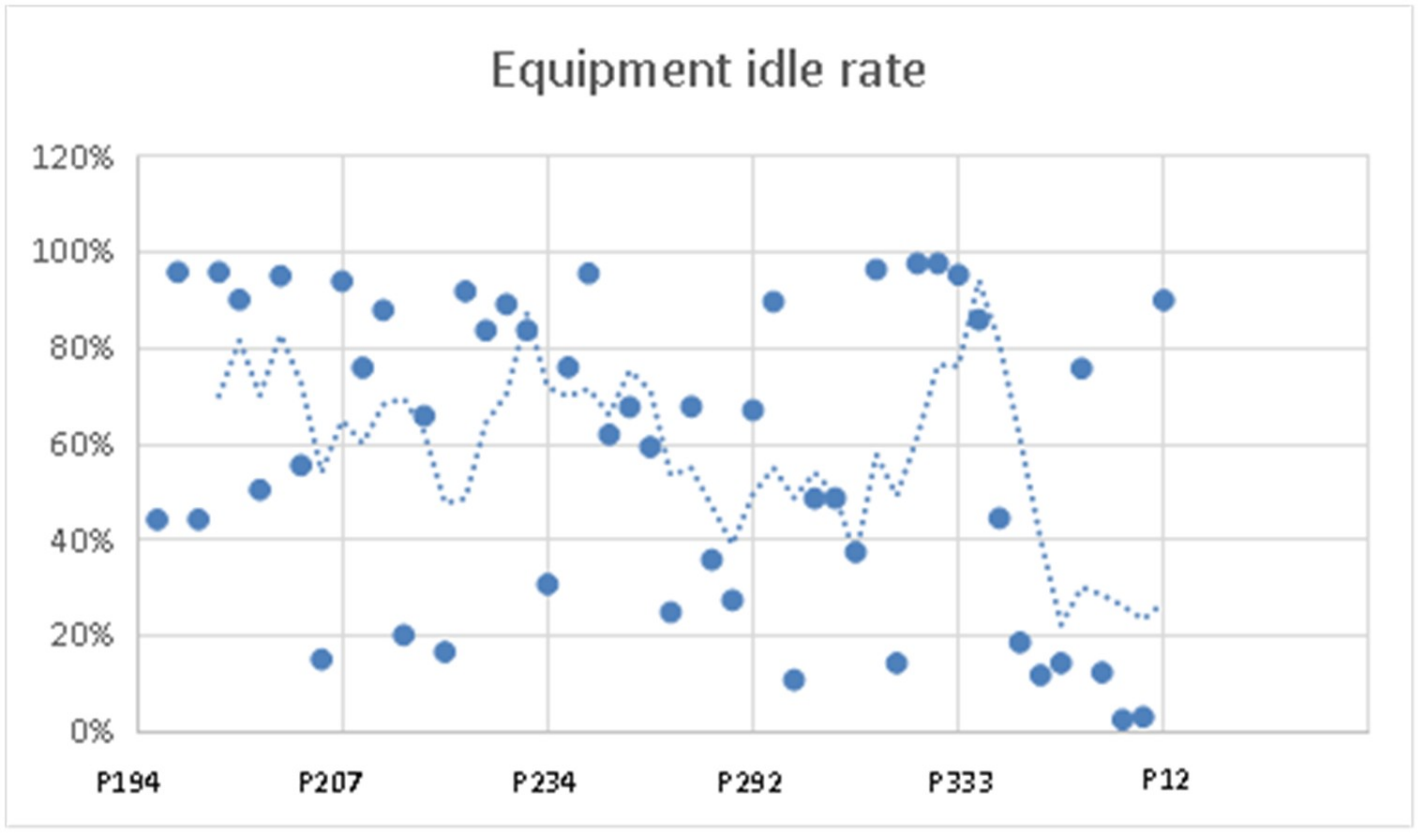
Equipment idle rate scatter plot and fitting curve graph.

1. Equipment idle rate
  Through statistical analyzing of the data with the module in FlexSim software, the proportion of different working states of different equipment in the entire production process can be clearly calculated, to obtain the utilization of different equipment. According to the initial simulation results (Table 8) of the working conditions of the production system, the idle rate of each device can be calculated as equipment idle rate = idle / state_since * 100%. With the moving average method to fit the change trend curve, a scatter plot of the idle rate of each device is drawn (Fig 12).
  From the Fig 12, three areas with improvement potential can be identified where the equipment idle rate is significantly higher, namely the drilling operation area (P199, P201, P207, P210, P223, P312), the lathe machining operation area (inner circle) (P195, P197, P224, P243, P322) and precision lathe processing operation areas (P227, P297, P324, P335).
2. Number of works in process production
  When the number of semi-finished products accumulated in a process is increasing, the equipment of the process is always in working status, and the processing capacity of the equipment is weakling, which will affect the subsequent processes, resulting in the production efficiency and production capacity of the entire production system to decline. However, because the number of works in process is difficult to count by simulation, the analysis is performed with the blockage rate of the equipment.
  According to the initial simulation results (Table 8) of the working condition of production system, the blocking rate of each device can be calculated as equipment blocking rate = blocked / state_since * 100%, which is shown in Table 9.

**Table 9:** Equipment blockage table.

Entity	Blockage time	Rate of blockage
Processor 282	6.47	0.02%
Processor 285	821.03	2.85%
Processor 345	6021.41	20.10%
Processor 9	18754.49	65.29%
Processor 10	21514.72	75.28%

It can be concluded from Table 9 that five processors have different degrees of blockage. Among them, three of the processors with significant higher rates of blockage are identified as bottlenecks, which are the processor 345 (20.10%), the processor 9 (65.29%) and processor 10 (75.28%). Through the analysis of the processing flow and time of the bearing production, the processor 345 is in the heat treatment / gear processing operation area, while the processor 9 and processor 10 are in the anti-corrosive area, the subsequent processing time of those two processors is 1440. We find that the main reason for the blockage is varying widely in processing time before and after, which cause an imbalance in the production line. Therefore, to mitigate this time difference, it is recommended to increase the number of equipment in the lathe processing operation area for subsequent processes.

#### Improvement strategy

The computer simulation optimization of discrete event dynamic system can overcome the difficulty of solving traditional production system equipment improvement and the limitation of the solution method, and generate results quickly and accurately, by utilizing its built-in data and flow analysis functions. From the analysis of the simulation results in Table 8, the production system of the bearing plant has an unbalanced equipment utilization, which causes an unbalanced production.

From the chapter of ‘Facilities layout improvement of bearing plant based on systematic layout planning (SLP) method’, we know that the layout of the bearing plant is unreasonable. Therefore, from the above perspective, the existing production system is improved to balance the utilization of different equipment, which in-turn improves the overall production efficiency and achieves the purpose of ameliorating the production system logistics.

We propose two strategies based on above analysis.

1. Increase / decrease the number of equipment
  According to the analysis the equipment idle rates in the three operating areas of the bearing production system are very high. Therefore, the equipment with idle rate higher than 80% is directly deleted in Table 8, which includes equipment P199, P201, P207, P210, P223, P312, P195, P197, P224, P243, P322, P227, P297, P324, P335. It not only saves a lot of costs, but also balances the production line. At the same time, according to the analysis of equipment blockage rate, it can be known that the blocking of processor 9 and 10 in the anti-corrosive area is due to the processing time of its subsequent process (processor 11) which is 1440. Therefore, the processor 1 and 2 which are same as processor 11 should be added. Similarly, the blockage of processor 345 in the heat treatment / gear processing operation area are also clogged because of the processing time of a subsequent process which is 1440. Hence, the processor 3 which is same as processor 346 should be added. For the blockage of processor 282, 285 in the lathe processing work area (outer ring), the same processor (processor 4) should be added due to fewer types of equipment.
2. Improve plant layout
  The plant has experienced the merger and the expansion of production processes and equipment. Due to the unreasonable layout of the production units, the logistics route of the workshop has a roundabout and crossover phenomenon, and the large-scale bearing plant is difficult to move, so a reasonable plant layout is very important. According to the analysis of the SLP method used in this paper, it is known that the layout of the workshop is unreasonable. The improved layout of the plant after the interchange of the assembly area, the work in process warehouse and the precision lathe processing operation area is shown in Fig 9.

### Improved model simulation and result analysis

**Fig 13:**
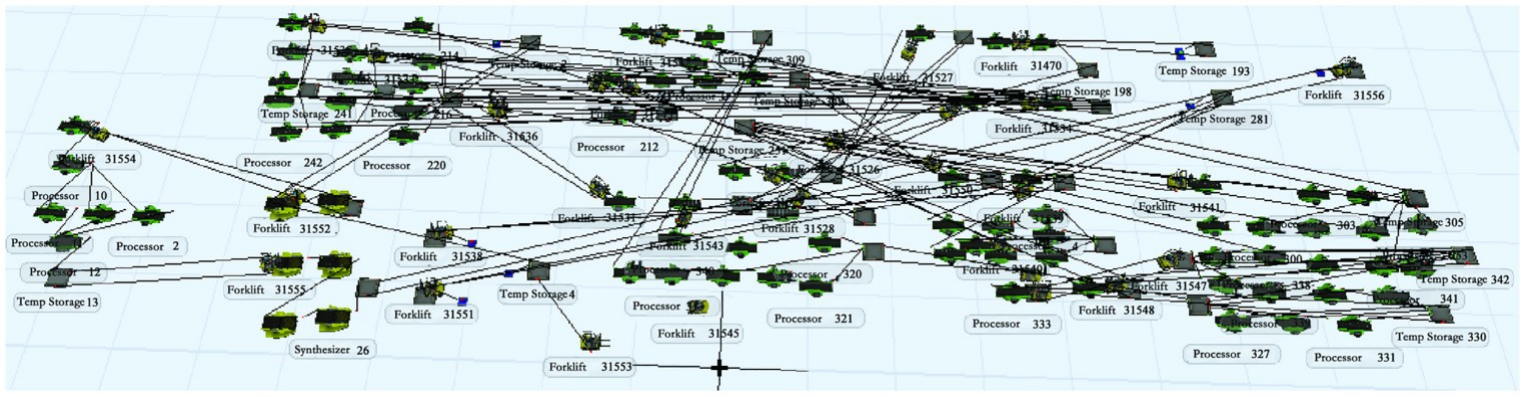
Simulation model of layout logistics system after bearing plant improvement.

The two methods proposed in the improvement strategy mainly aim at the three problems, imbalanced utilization of equipment in the production logistics system of the bearing plant, serious accumulation of work in progress (high equipment blocking rate), and unreasonable layout of the plant. This section combines these two improvement strategies to reasonably form a complete improvement scheme and re-establish a simulation model of the bearing plant production system (Fig 13).

After the establishment of the improved FlexSim simulation model of the bearing plant production logistics system, the parameters of each processing equipment are set. Other simulation conditions such as the shift calendar are unchanged. The end state simulation method is still used. After the simulation runs 28800, a comparative analysis of the equipment idle rate and blockage rate of each processing step after improvement is obtained, as shown in Tables 10 and 11.

**Table 10:** Comparison of equipment idle rates before and after improvement.

Entity	Free time before optimization	Idle rate before optimization	Idle time after optimization	Idle rate after optimization	Entity	Free time before optimization	Idle rate before optimization	Idle time after optimization	Idle rate after optimization
Processor 195	26258.91	95.84%	0	0%	Processor 227	24014.63	83.78%	0	0%
Processor 197	26318.91	95.85%	0	0%	Processor 243	27511.21	95.57%	0	0%
Processor 199	25892.85	90.08%	0	0%	Processor 297	25733.19	89.68%	0	0%
Processor 201	27372.85	95.05%	0	0%	Processor 312	27584.79	96.38%	0	0%
Processor 207	27021.3	93.95%	0	0%	Processor 322	27925.22	97.62%	0	0%
Processor 210	25198.61	87.93%	0	0%	Processor 324	28018.62	97.63%	0	0%
Processor 222	26333.72	91.84%	21766.7	75.61%	Processor 333	27420.15	95.27%	27278.58	94.78%
Processor 223	24023.72	83.70%	0	0%	Processor 335	24517.68	85.91%	0	0%
Processor 224	25615.37	89.14%	0	0%					

**Table 11:** Comparison of blocking situation before and after improvement.

Entity	Block time before optimization	Initial blockage rate	Block time after optimization	Optimized blocking rate	Blockage reduction percentage	entity	Block time before optimization	Initial blockage rate	Block time after optimization	Optimized blocking rate	Blockage reduction percentage
Processor 282	6.4663	0.023%	0	0.00%	100.00%	Processor 9	18754.5	65.29%	0	0.00%	100.00%
Processor 285	821.03	2.85%	0	0.00%	100.00%	Processor10	21514.7	75.28%	0	0.00%	100.00%
Processor 345	6021.41	20.10%	0	0.00%	100.00%						

According to the data in Table 10, the high idle processing equipment in the bearing plant production system have been improved and reduced from 17 to only 2. This situation may be caused by the short processing time of the equipment at these stations, the low utilization of the equipment, and the failure to give full play to its original production capacity. Therefore, the imbalance in the production system is caused.

According to the data in Table 11, it can be seen that the clogging rate of the equipment is reduced by 100%. While there is no clogging of the equipment in the ameliorate production system, it is indicated that the improvement scheme proposed in this paper meet the demand for production capacity, eliminate the blockage of the production line, improve equipment utilization, and make the production system more balanced. Therefore, after the improvement, the blockage of the equipment and the idle rate of the equipment are greatly improved, and the production line is more balanced.

## Conclusion

Whether the plant logistics planning of a manufacturing enterprise is reasonable has a great impact on the production efficiency and profitability of an enterprise, and directly affects the quality and cost of the production. Based on the research in this paper, it can be known that the proposed improvement method can solve the problems of unbalanced equipment utilization, bottleneck, unreasonable plant layout, effectiveness of interaction between simulation model and actual production system etc. This study investigates the status of a large wind power bearing production plant, with collection of relevant information and data and its application to the SLP method to re-plan the layout of plant facilities. In addition, in order to find the bottlenecks that restrict the entire plant production logistics system, this paper establishes a bearing manufacturing process model with Petri net method, and verifies the validity of the theoretical model with data such as the number of equipment and processing time in the production process. This modeling method has the advantages of simple and intuitive, easy to understand, and strict mathematical representation. However, it cannot meet the requirements of data types and data quantities required for numerical simulation. Therefore, the advantages of data interface, modular modeling, and dynamic display of statistical performance parameters are provided for the simulation software. It combines Petri net and FlexSim simulation to establish a model mapping relationship between the two. Synthesizing the advantages of Petri net and FlexSim simulation to analyze the production system can better achieve the improvement simulation of the system. Followed by the analysis of problems in blockage and imbalance in the production, and the assembly of the bearing based on the simulation results, suggestions for changing the number of equipment are put forward. With the combined plant layout plan obtained by the SLP method, a complete improvement plan is formed. Subsequently, the production system blockage and production imbalance problem are solved and alleviated by re-simulation and comparative analysis. Moreover, the effectiveness and rationality of the proposed scheme is verified in this paper which provides some reference for the improvement of the plant layout of relevant enterprises. In the future study, researchers should focus on the theoretical minimum process equipment calculation, combining the characteristics of the bearing plant production system, and adopting the method of station reorganization to rationally adjust the process, reduce the number of equipment, and increase the production capacity of the entire production system.
